# Supplementary material for: The genome sequence of an ichneumonid wasp, Pimpla contemplator (Muller, 1776)
Source: Wellcome Open Res. 2024 Nov 5;9:647. [Version 1] doi: 10.12688/wellcomeopenres.23303.1 (PMC11628944; doi:10.12688/wellcomeopenres.23303.1)
Supplement: Peer review history

# Reviewer report: Reviewer response for version 1

**DOI:** 10.21956/wellcomeopenres.25685.r110338

**Published:** 2024-12-09

**Authors:** Avinjikkattu Parambil Ranjith

**Affiliations:** 1 Chulalongkorn University, Bangkok, Bangkok, India

I have reviewed the manuscript and don't have any comments on this version of manuscript. I only checked the taxonomic part of the manuscript since I am not an expert to comment on the molecular data. I found only a minor error on the author name of the species. It should be Müller not Muller. Please accept the manuscript as it's current form.

Are sufficient details of methods and materials provided to allow replication by others?

Yes

Is the rationale for creating the dataset(s) clearly described?

Yes

Are the datasets clearly presented in a useable and accessible format?

Yes

Are the protocols appropriate and is the work technically sound?

Yes

Reviewer Expertise:

Taxonomy and systematics of parasitoid wasps

I confirm that I have read this submission and believe that I have an appropriate level of expertise to confirm that it is of an acceptable scientific standard.

# Reviewer report: Reviewer response for version 1

**DOI:** 10.21956/wellcomeopenres.25685.r112564

**Published:** 2024-12-06

**Authors:** Alice Dennis

**Affiliations:** 1 University of Namur, Namur, Belgium

This report describes the genome of a male individual of the parasitoid wasp *Pimpla contemplator.* This system has the advantage that males are haploid, thus simplifying the assembly a bit. The genome was assembled using PacBio Hifi reads, with approx 230x coverage. Scaffolding was performed with HiC, including manual curation. The authors report >92% of the genome can be assigned to 18 scaffolds, thus nearing chromosome level for this assembly. The mitochondrial genome was also assembled. Unfortunately, no link is made to an annotation.

 This is a sound result and useful resource. I have one broad suggestion to improve this report:

 The methods are very detailed, but lack a bit of insight into their actual purposes. For example, blobtools is described well but there is no mention of screening for contamination. I suggest revisiting the methods to see if there are other places to give a small amount of insight into the purpose of the tools.

 Under Assembly this is most needed (e.g. pg8). In general, this focuses on the tools that were used, but not what they were used for.

     - What aspect of Gfastats was used? It gives many statistics but I guess it was used to obtain some key criteria

     - For the mitogenome assembly, what species/ file was used as input. Did you use the raw reads, or the assembly? What did OATK do?

     - For manual curation, you refer to another manuscript, but should include a few details here.

 Typigraphical issues

 -"Basically" used twice in beginning of Background. Suggest rephrasing

 -Also in Background, perhaps explain what crepuscular means?

 -Page 8, under "Assembly", double period

Are sufficient details of methods and materials provided to allow replication by others?

Partly

Is the rationale for creating the dataset(s) clearly described?

Yes

Are the datasets clearly presented in a useable and accessible format?

Yes

Are the protocols appropriate and is the work technically sound?

Yes

Reviewer Expertise:

Evolution, genomics, and molecular ecology of nonmodel invertebrates

I confirm that I have read this submission and believe that I have an appropriate level of expertise to confirm that it is of an acceptable scientific standard, however I have significant reservations, as outlined above.

# Reviewer report: Reviewer response for version 1

**DOI:** 10.21956/wellcomeopenres.25685.r110337

**Published:** 2024-11-20

**Authors:** Ariel D Chipman

**Affiliations:** 1 Hebrew University of Jerusalem, Jerusalem, Jerusalem District, Israel

This is a report of the genome sequence of the parasitoid wasp *Pimpla contemplator.* The identification of the specimen is reliable. The methods and analyses are technically sound and fairly standard. The work complies with the expected standards of a report of this type. I have no problems or criticism of the manuscript.

Are sufficient details of methods and materials provided to allow replication by others?

Yes

Is the rationale for creating the dataset(s) clearly described?

Yes

Are the datasets clearly presented in a useable and accessible format?

Yes

Are the protocols appropriate and is the work technically sound?

Yes

Reviewer Expertise:

Arthropod evolution, comparative genomics.

I confirm that I have read this submission and believe that I have an appropriate level of expertise to confirm that it is of an acceptable scientific standard.
